# SYNGAP1: Mind the Gap

**DOI:** 10.3389/fncel.2016.00032

**Published:** 2016-02-15

**Authors:** Nallathambi Jeyabalan, James P. Clement

**Affiliations:** ^1^Narayana Nethralaya Post-Graduate Institute of Ophthalmology, Narayana Nethralaya Foundation, Narayana Health CityBangalore, India; ^2^Neuroscience Unit, Jawaharlal Nehru Centre for Advanced Scientific ResearchBangalore, India

**Keywords:** SYNGAP, synaptic plasticity, intellectual disability, autism spectrum disorders, learning and memory, neurodevelopmental disorders

## Abstract

A cardinal feature of early stages of human brain development centers on the sensory, cognitive, and emotional experiences that shape neuronal-circuit formation and refinement. Consequently, alterations in these processes account for many psychiatric and neurodevelopmental disorders. Neurodevelopment disorders affect 3–4% of the world population. The impact of these disorders presents a major challenge to clinicians, geneticists, and neuroscientists. Mutations that cause neurodevelopmental disorders are commonly found in genes encoding proteins that regulate synaptic function. Investigation of the underlying mechanisms using gain or loss of function approaches has revealed alterations in dendritic spine structure, function, and plasticity, consequently modulating the neuronal circuit formation and thereby raising the possibility of neurodevelopmental disorders resulting from synaptopathies. One such gene, *SYNGAP1* (Synaptic Ras-GTPase-activating protein) has been shown to cause Intellectual Disability (ID) with comorbid Autism Spectrum Disorder (ASD) and epilepsy in children. SYNGAP1 is a negative regulator of Ras, Rap and of AMPA receptor trafficking to the postsynaptic membrane, thereby regulating not only synaptic plasticity, but also neuronal homeostasis. Recent studies on the neurophysiology of SYNGAP1, using *Syngap1* mouse models, have provided deeper insights into how downstream signaling proteins and synaptic plasticity are regulated by SYNGAP1. This knowledge has led to a better understanding of the function of SYNGAP1 and suggests a potential target during critical period of development when the brain is more susceptible to therapeutic intervention.

## Introduction

The brain is the center of the nervous system and is the most complex organ in the body. All day-to-day activities including executive decisions, memories, emotions, and cognitive tasks are mediated by the cerebral activity. Apart from coordinating the ability to smell, touch, hear, taste, and see, the brain enables people to form words, perform mathematical calculations, communicate using different languages, grasp and appreciate music, make decisions, organize and plan everyday activities and above all, imagine. Therefore, normal development of brain is imperative for performing these and other essential functions. A cardinal feature of early stages of human brain development centers on the sensory, cognitive, and emotional experiences that shape neuronal-circuit formation and refinement. Consequently, alteration in any of these features accounts for many psychiatric and neurological disorders (Spooren et al., 2012; Kroon et al., 2013).

The human brain consists of 86 billion neurons and 85 billion non-neuronal cells (Azevedo et al., 2009), which play a vital role in information processing and transmission in the form of electrical signals through specialized junctions called synapses. Neuroscientists have made great progress in unraveling the cellular and molecular mechanisms of dendritic spine synapse formation and function, which is considered as one of the most remarkable developments in biology in the last three decades. A precise control of synaptic development and neuronal connectivity has been found to be necessary for normal brain development. Conversely, abnormal dendritic spine morphology and function can lead to disruption of neuronal circuits, and consequently can result in various psychiatric and neurodevelopmental disorders (NDDs; Melom and Littleton, 2011).

Altered dendritic spine function and neuronal circuit formation account for one of the major underlying mechanisms of Intellectual Disability (ID) and Autism Spectrum Disorder (ASD; Chechlacz and Gleeson, 2003; Kroon et al., 2013), which are often co-diagnosed in young children with NDDs and affect 1–3% of the general population. Due to high rates of co-morbidity of these NDDs, it has been broadly hypothesized that ID and ASD share common neurodevelopmental pathologies that lead to various behavioral and cognitive symptoms that define these disorders. The underlying cause of these NDDs are believed to be mutations in genes, parental drug use and aging process, viral infections and other environmental factors (van Spronsen and Hoogenraad, 2010).

Recent evidences from many animal models of ID and ASD suggest that mutations that cause NDDs occur in genes encoding the proteins that regulate synaptic function and/or structure (Boda et al., 2002; Bear et al., 2004; Ramocki and Zoghbi, 2008; Südhof, 2008; Gauthier et al., 2011; Penzes et al., 2011). Mutations in many of these single-genes are the major cause of syndromic and non-syndromic ID (NSID; Bhakar et al., 2012; Zoghbi and Bear, 2012). The most common single-gene mutations in ASD with ID are associated with Fragile X syndrome (FXS; *FMRI*), Tuberous Sclerosis (*TSC1*, *TSC2*), Angelman Syndrome (*UBE3A*), Rett Syndrome (*MECP2*), and Phlean-McDermid syndrome (*SHANK3*). Rare mutations in single-genes, such as those encoding for Neuroligin (*NLGN3, NLGN2*) and Neurexin (*NRXN1*), are also implicated in ID and ASD. These genes are just a few of many implicated in NDDs, suggesting that highly penetrant mutations of genes play an important role in regulating synaptic function. Heterozygous mutation in *SYNGAP1* cause ID and ASD, and whose product is now established as a major regulator of synaptic function.

**Figure 1:**
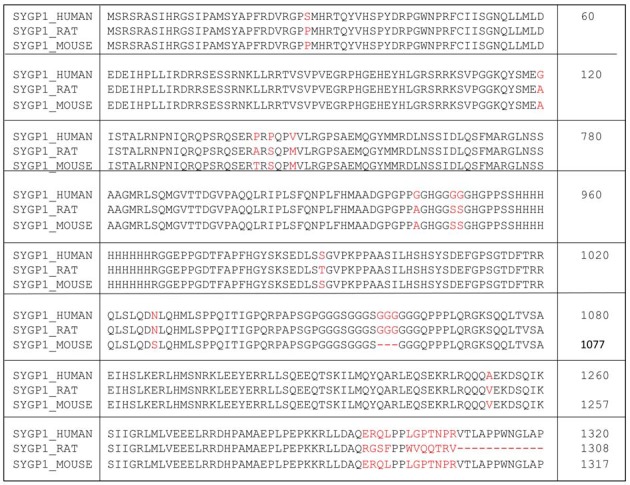
**Amino acid sequence of human SYNGAP1 and its difference with mouse SYNGAP1.** Amino acid sequence differences of SYNGAP1 between *Homo* sapiens (SYGP1_Human), *Rattus rattus* (SYGP1_RAT) and *Mus musculus* (SYGP1_MOUSE). Variations in the sequences were indicated in red colored fonts. Dashed line indicates empty sequences.

Numerous studies have shown that a major share of dendritic spine synapses utilize the excitatory neurotransmitter, glutamate, to activate N-methyl D-aspartate receptors (NMDARs), which are associated with a vast array of transmembrane proteins, scaffolding proteins and many signaling proteins (Pèrez-Otaño and Ehlers, 2004; Lau and Zukin, 2007; Kerchner and Nicoll, 2008; Lai and Ip, 2013; Fan et al., 2014). SYNGAP1, is a downstream component of NMDAR-associated signaling complex that negatively regulates activation of small GTP-ase (Ras- and Rap-GAP) and of α-amino-3-hydroxy-5-methyl-4-isoxazolepropionic acid receptor (AMPAR) trafficking to excitatory postsynaptic membrane (Rumbaugh et al., 2006; Huang, 2009; Walkup et al., 2015). *SYNGAP1* is a ~140 kDa protein located on Chromosome 6p21.3^1^ (Figure 1; Husi et al., 2000). Phosphorylation of SYNGAP1 is regulated by CaMKII, which reduces SYNGAP1’s control of Ras-GTPase, leading to Ras activation by increasing the GTP-bound form of Ras. It is now established as a major signaling protein that plays a pivotal role in regulating fundamental molecular changes in dendritic spine synaptic morphological and functional modifications. Moreover, mutations in SYNGAP1 are established as relevant for human pathology, because they have been associated with ID comorbid with ASD in children (Hamdan et al., 2009, 2011a,b, 2014; Gauthier et al., 2011; Berryer et al., 2013).

Until a few years ago, the neurophysiological mechanism that causes ID in patients with *SYNGAP1* mutation was not clear. Using mouse models of *Syngap1* heterozygous mutations (*Syngap1^−/+^*), several labs have shown that 50% reduction of the level of SYNGAP1 is sufficient to cause significant increases in the presence of mushroom-shaped dendritic spines during early stages of development resulting from a lack of inhibition of Ras-GTPase, which in turn allows more AMPARs to be transported to the postsynaptic membrane. Moreover, it has been shown that in *Syngap1^−/+^* models certain critical periods of neuronal growth and maturation are disrupted, leading to developmental brain disorders, that in turn cause cognitive and social dysfunctions (Guo et al., 2009).

## *Syngap1* Expression, Functional Domains, and Isoforms

SYNGAP1 is a ~140 kDa protein, first characterized by Chen et al. (1998) using a mouse model of *Syngap1^−/+^* mutation and followed by Kim et al. (1998) who developed a truncated form of SYNGAP1 using yeast two-hybrid system (See Table 1 for Historical perspective). Based on these studies, it can be understood that the N-terminal half of SYNGAP1 has a Ras-GAP domain, along with the region that is loosely homologous to Pleckstrin homology and a C2 domain which is potentially involved in binding of Ca^2+^and phospholipids. Interestingly, the alignment of GAP domain of SYNGAP1 with other Ras-GAPs suggests that the amino acids in GAP domain are vital for interaction with Ras and for the stimulation of Ras-GTPase activity (Chen et al., 1998; Kim et al., 1998). Given the Ras-GAP-interacting domain of a newly discovered protein and its presence in excitatory synapses (Chen et al., 1998; Kim et al., 1998), this protein was termed SYNGAP1 (Synaptic Ras-GAP activator protein). Moreover, studies have shown that SYNGAP1 is expressed only in brain tissue and not in other tissues (Chen et al., 1998; Kim et al., 1998). In the brain, it is primarily expressed in the excitatory neurons, where it is localized to synapses. On the contrary, SYNGAP1 was absent in inhibitory neurons. Chen et al. (1998) have shown that the carboxyl terminal tail of SYNGAP1 interacts with postsynaptic density protein (PSD-95), as confirmed by their coprecipitation (Kim et al., 1998). In addition, Kim et al. (1998) have shown that the C-terminal half consists of a repeat of 10 histidines that may be involved in metal chelation, several potential serine and tyrosine phosphorylation sites and a T/SXV motif necessary for interaction with SAP102 and PSD-95. This study further suggested that SYNGAP1 is a cytosolic protein without a signal peptide or any transmembrane domain (Kim et al., 1998).

**Table 1:** **Historical perspective of major findings of *SYNGAP1***.

Observation/findings of SYNGAP1	Referrences	Model/samples
First *Syngap1* Het mouse model	Chen et al. (1998)	Mouse
Binds to CaMKII /PSD95	Chen et al. (1998)	Mouse
	Kim et al. (1998)	Yeast two-hybrid hippocampal cDNA library
Amino acid sequences and molecular weight	Chen et al. (1998)	Mouse
	Kim et al. (1998)	Yeast two-hybrid hippocampal cDNA library
	Husi et al. (2000)	Mouse
	McMahon et al. (2012)	Mouse
Domain structure of SYNGAP1 and Isoforms	Chen et al. (1998)	Mouse
	McMahon et al. (2012)	Mouse
Localized in excitatory neurons	Chen et al. (1998)	Mouse
	Kim et al. (1998)
	Kim et al. (1998)	Yeast two-hybrid hippocampal cDNA library
*Syngap1* Homozygous mice die within a week	Komiyama et al. (2002)	Mouse/Primary neuronal culture
	Kim et al. (2003)	Mouse/cortical culture
Synaptic transmission and LTP	Komiyama et al. (2002)	Mouse/Primary neuronal culture
	Kim et al. (2003)	Mouse/cortical culture
Learning and Memory deficits	Komiyama et al. (2002)	Mouse/Primary neuronal culture
Altered ERK, Ras, Rac p-Cofilin	Komiyama et al. (2002)	Mouse/Primary neuronal culture (ERK)
	Carlisle et al. (2008)	Mouse/Hippocampi neuronal culture
Dendritic spine structure	Vazquez et al. (2004)	Mouse/primary neuronal culture
	Carlisle et al. (2008)	Mouse/Hippocampi neuronal culture
Cognitive and social dysfunction	Guo et al. (2009)	Mouse
	Muhia et al. (2010)	Mouse
Intellectual disability in children	Hamdan et al. (2009)	Human
Prematuration of dendritic spines	Clement et al. (2012)	Mouse
	Aceti et al. (2015)	Mouse (*in vivo*)
Altered critical period of plasticity	Clement et al. (2013)	Mouse

*This table summarizes the major findings/observations of function of SYNGAP1 by various groups*.

**Figure 2:**
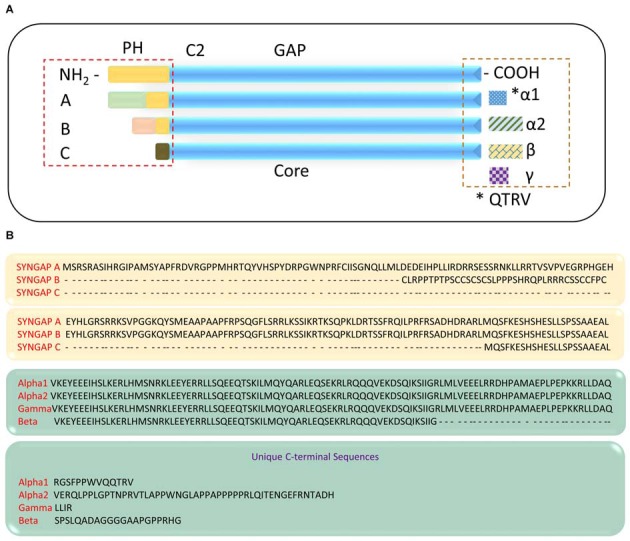
**SYNGAP1 isoforms arising from alternate splicing. (A)** A schematic illustration of potential SYNGAP1 isoforms, which vary in both N- and C-termini. **(B)** Amino acid sequences that are unique to different N- and C-termini isoforms identified by mass spectrometry (McMahon et al., 2012).

Studies have shown that functionally distinct proteins may be produced via regulated alternate splicing of mRNA (Lipscombe, 2005; Li et al., 2007; Grabowski, 2011; Raj and Blencowe, 2015). It is evident that *Syngap1* is a complex gene that gives rise to multiple protein domains. This further implies that *Syngap1* may be spliced differentially, which can lead to different isoforms. Indeed, Chen et al. (1998) showed that two splice variants were observed, one at the amino terminus and one at carboxyl terminus, which further encode four variants with molecular weights of 134, 137, 140 and 143 KDa. The amino acid terminal contains a putative Pleckstrin homology (PH) domain (Chen et al., 1998), which may attach the protein to the membrane (Lemmon and Ferguson, 2000). In support of this finding, a recent study has identified distinct isoforms of SYNGAP1 (Figure 2), differing in their N- and C-terminals (McMahon et al., 2012). The existence of different isoforms was further confirmed when anti-SYNGAP1 antibody recognized doublet or triplet of proteins at 130 kDa only in the brain, with no detection of SYNGAP1 and its isoforms in any other parts of the body such as kidney, heart or lung (Chen et al., 1998; Kim et al., 2003). Each isoform contains a central GAP domain to regulate the activity of GTPase in small GTPases such as Ras and Rap. Three distinct *Syngap1* isoforms, SYNGAP A, B and C, differing in their N-termini arising from different promoter regions have been identified (McMahon et al., 2012). A and B isoforms contain unique peptide sequence and a complete PH domain, whereas isoform C is a shorter, truncated protein with no unique peptide sequence and no PH domain. Furthermore, SYNGAP A, B, and C isoforms can be subdivided based on transcription start sites (A1–A11; B1; C1–C8). To determine whether the multiple promoters were also present in humans, a sequence comparison with mouse and rat revealed a highly conserved regions with no predicted functional moieties (McMahon et al., 2012). Finally, alternate splicing of *Syngap1* mRNA leads to multiple isoforms of C-termini, designated as α, β and γ. Of these, the most studied C-terminus isoform is SYGNAP1 α1, which contains the PDZ-binding domain and mediates binding to scaffolding proteins of PSD.

The expression of various genes that encode proteins regulating synaptic formation and function are shown to reach their peak level of expression during early stages of development. Moreover, several of these proteins have recently been implicated in ID (State and Levitt, 2011; State and Sestan, 2012). Indeed, expression of SYNGAP1 in neurons reaches its peak 14 days after birth, i.e., at Postnatal day 14 (PND 14 days) in rodents (Kim et al., 1998; Clement et al., 2012), but steadily decreases to adult level by 2 months of age (Porter et al., 2005). Further, SYNGAP1 is highly expressed in hippocampus and cortex, and less in striatum (Komiyama et al., 2002). The expression of SYNGAP1 was reduced by 50% in *Syngap1^−/+^* mice (Komiyama et al., 2002; Kim et al., 2003; Clement et al., 2012). However, no abnormal gross development of brain was observed in *Syngap1^−/+^* mice (Kim et al., 2003).

It is well known that development and synaptic activity plays a major role in differential splicing of genes involved in synaptic function. The N-termini SYNGAP1 isoforms, A and B had a pattern of regulation during development (gradual increase in expression till PND14), while isoform C was expressed at very low level till PND14 (McMahon et al., 2012). However, all three isoforms, A, B, and C reached their peak expression at PND14. Further, it is shown that Syngap B and C were up-regulated two-fold after 4 h of bicuculline treatment, whereas SYNGAP A was down-regulated (McMahon et al., 2012). These changes were inhibited in the presence of tetrodotoxin. This confirms that differential splicing of *Syngap1* occurs based on neuronal activity, which leads to opposing functional effects of *Syngap1* isoforms; SYNGAP A had silencing effect, while SYNGAP B and C had positive effect on synaptic transmission and strength. Unlike N-termini isoforms, the protein levels of C-termini isoforms did not change when stimulated with bicuculline. Importantly, it is not clear from previous studies which combination of N- and C- termini isoforms exists in neurons. Using isoform-specific antibodies, it is clear that both C-terminal isoforms exist in hippocampus and cortex (McMahon et al., 2012; Yang et al., 2013) and that α2-containing isoforms localize to PSDs, although they do not contain a QTRV region to bind to PDZ domain. This could be because, when non-phosphorylated CaMKII is inactive at rest, both isoforms are localized within PSD core and α1 binding to PSD-95 serves a distinct function, that of blocking the other portions from binding to PSD-95. Furthermore, upon activation of NMDARs, both α1 and α2 isoforms move out of the PSD core, but this change was reversed within 30–45 min following the NMDAR activation.

This movement of SYNGAP1 α1 and α2 out of PSD core could have two significant effects. The first major effect could be a displacement of GAP activity and thus induces activity-dependent synaptic modification (Yang et al., 2013; Araki et al., 2015). The second major effect of SYNGAP1 α1 and α2 isoforms moving out of the PSD core is to create an empty slot for the association of an AMPAR (Yang et al., 2013), thereby regulating synaptic strength. In fact, overexpression of SYNGAP1 α1 isoform reduces AMPAR-mediated miniature excitatory postsynaptic currents (mEPSCs), whereas overexpression of SYNGAP1 α2 isoform enhances mEPSCs. Nevertheless, these studies illustrate the fact that different isoforms exert opposing effects on synaptic strength and that both full peptide sequences, N- and C-terminal, and their isoforms must be considered when examining the functional properties of the protein.

## *Syngap1* Mutations in intellectual Disability

ID, formerly characterized as Mental Retardation, is defined by three criteria: (a) an intelligent quotation of less than 70; (b) limitations in two or more adaptive behaviors such as communication, self-care and social skills, community use, self-direction, health, and safety; and (c) evidence that the mental manifestations began before the age of 18 (van Bokhoven, 2011). ID comprises a diverse collection of syndromic and non-syndromic disorders. Unlike NSID, which is characterized by intellectual deficits as the only clinical feature, syndromic ID patients typically exhibit other abnormal clinical features, such as cranial, facial, and skeletal dysmorphisms. Major causes of ID and ASD stem from mutations of genes encoding for proteins that are critical regulators of synaptic function. *De novo* mutations in individual genes explain an important aspect of NSID characterized by the absence of associated morphologic, radiologic and metabolic features, as opposed to ID associated with more complex chromosomal aberrations (Ropers and Hamel, 2005). The genetic factors involved in NSID are not clearly known. So far, 29 X-linked and five autosomal recessive genes have been identified by linkage and cytogenetic analysis (Bienvenu and Chelly, 2006; Chelly et al., 2006; Gécz et al., 2009). In addition, mutations in autosomal dominant genes are still to be identified in NDDs, as ID and ASD result in lower reproductive probability, which further reduces the chances of identifying families that are open to linkage analysis. Yet, *de novo* mutations are the most commonly recognized cause of ID, which suggests that monoallelic lesions are sufficient to cause this disorder. In fact, studies have shown that one to three *de novo* mutations per zygote affect amino acid sequences (Crow, 2000a,b, 2006).

In the past decade, novel autosomal *de novo* mutations were identified in genes encoding for proteins involved in synaptic plasticity. In relation to that, recent studies from children have shown that *de novo* truncating mutations in *SYNGAP1* cause NSID (Hamdan et al., 2009, 2011a,b, 2014; Berryer et al., 2013). Hamdan et al. (2009) identified protein-truncating *de novo* mutations in *SYNGAP1* in 3 of 94 patients with NSID. These patients ranged from 4–11 years and had similar clinical features as described in the Mullen Scale of Early Learning and the Vineland Adaptive Behavior Scales (Hamdan et al., 2009). Of those three patients, two patients were heterozygous for nonsense mutations, while the third patient was heterozygous for a frame-shift mutation starting at codon 813, producing a premature stop codon at 835. All these children were born to non-consanguineous parents. During early stages of development, these children presented with hypotonia and global delay of development with the onset of walking at 2-years. Apart from these defects, two of these children had presented with tonic-clonic and myoclonic seizures.

In order to further explore the association of *SYNGAP1* to ID, particularly to understand whether patients with epilepsy and ASD had *SYNGAP*1 *de novo* mutations, Hamdan et al. (2011a) sequenced exons of *SYNGAP1* from additional cohorts of patients. *De novo* out-of-frame deletions were identified in two patients with NSID presented with microcephaly and generalized epilepsy. The authors also described a *de novo* splicing mutation in a patient with autism that had not acquired microcephaly or epilepsy (Refer to Table 2). Moreover, missense mutations in *SYNGAP1* were detected in three patients. Surprisingly, all these patients were born to non-consanguineous parents. Furthermore, other studies (Krepischi et al., 2010; Zollino et al., 2011; Writzl and Knegt, 2013) have identified *de novo* microdeletions in chromosome region 6p21.3 in patients with ID, epilepsy and severe language impairment. Therefore, it is evident from the studies that heterozygous mutations in *SYNGAP1* are the major cause of NSID.

**Table 2:** **Clinical features observed in patients with *SYNGAP1* heterozygous mutation**.

Patient no	Gene	DNM	Age	Sex	ID	Epilepsy	Cranial MRI/CT
1	SYNGAP1	p.Lys138X	4 yrs. 5 months	F	++/+++	GN	Normal/ND
2	SYNGAP1	p.Arg579X	5 yrs. 10 months	F	++/+++	GN	Normal/ND
3	SYNGAP1	p.Leu813ArgfsX23	12 yrs. 10 months	F	++/+++	–	ND/Normal
4	STXBP1	p.Arg388X	15 yrs.	F	+++	PC	Normal/Normal
5	STXBP1	c.169–1G>A	27 yrs.	F	+++	PC	ND/Normal
6	SHANK3	c.601–1G>A	15 yrs.	M	+	–	ND/Normal
7	KIF1A	p.Thr99Met	3 yrs. 5 months	F	+++	–	Atrophy/ND
8	GRIN1	p.Glu662Lys	10 yrs.	F	++	–	ND/Normal
9	GRIN1	p.Ser560dup	7 yrs. 6 months	M	+++	PC	Normal/ND
10	EPB41L1	p.Pro854Ser	6 yrs.	M	+++	–	Normal/ND
11	CACNG2	p.Val143Leu	8 yrs.	M	++	–	Normal/ND
12	KIFC1, PHF1, CUTA, SYNGAP1	–	6 yrs.	M	++	–	Normal/ND
13	SYNGAP1, CUTA, PHF1	–	5 yrs.	F	+++	GN	Normal/ND
14	SYNGAP1, CUTA, PHF1	–	9 yrs.	M	++	PC	Normal/ND

*Summary of clinical features observed in patients with SYNGAP1 heterozygous mutation. ID Scale: + denotes mild, ++ moderate, +++ severe. Abbreviations used ND, not determined; PC, Partial complex epilepsy; GN, Generalized epilepsy. These features are based on different sources (Hamdan et al., 2009, 2011a,b, 2014; Krepischi et al., 2010; Zollino et al., 2011; Berryer et al., 2013; Writzl and Knegt, 2013)*.

## *Syngap1* in Schizophrenia

Although several studies have shown the role of *Syngap1* mutations in neurodevelopmental disorders, a little is known about its relevance to schizophrenia. Converging evidence suggest that dysfunction of NMDARs and the signaling complex associated with them is now considered to be one of the major causes of schizophrenia (Belsham, 2001). Hypofunction of NMDARs was first implicated when reduced concentration of glutamate were found in the cerebrospinal fluid of patients with schizophrenia (Kim et al., 1980). The alteration in the function of activation-ready NMDAR complexes localized in the PSD can lead to a defect in downstream signaling pathways. It has been shown that a major function of SYNGAP1 is to transduce the activation of synaptic NMDA receptors to a biochemical signal that is necessary for proper neuronal function. Therefore, SYNGAP1 and its interacting proteins may be abnormal in patients with schizophrenia.

A study by Funk et al. (2009) has shown that SYNGAP1 and its interacting proteins, such as PSD95, were reduced in patients with schizophrenia. Interestingly, patients who were non-medicated for 6 weeks prior to the time of their death showed decreased levels of SYNGAP1 compared to medicated patients. A similar observation was made for SYNGAP1-interacting proteins such as PSD-95. This study hypothesized that *Syngap1^−/+^* are associated with schizophrenia-like behavioral phenotypes. Indeed, reduced expression of *SYNGAP1* results in abnormal behaviors that are strikingly similar to that reported in mice with reduced NMDAR function (Guo et al., 2009). This suggests that proteins downstream of NMDAR, including SYNGAP1, participate in a common pathway that may be dysfunctional in people with schizophrenia. However, other studies (Hamdan et al., 2009) did not find any *de novo* mutations, splicing or truncating, in their patients with schizophrenia. As the sample number of patients with schizophrenia studied in their work is low, more samples are needed to confirm the role of SYNGAP1 Het in schizophrenia.

## Mouse Models of *Syngap1*

The recent advances in genomic science and the development of transgenic technology in mice have advanced research into the effect of monoallelic mutations in genes that are associated with synaptic transmission and neuronal circuit formation. *SYNGAP1* is highly conserved across species (McMahon et al., 2012), which has allowed for the development of different animal models of *Syngap1^−/+^* mice (Komiyama et al., 2002; Kim et al., 2003; Vazquez et al., 2004; Muhia et al., 2010). The *SYNGAP1* mouse ortholog, *Syngap1*, is located on chromosome 17^2^. Interestingly, *Syngap1^−/−^* mutant mice do not survive for more than a week (Kim et al., 2003). This is due to increased levels of caspase-3 activation in *Syngap1^−/−^*, which suggests that apoptosis is enhanced by reduction of SYGNAP1 (Knuesel et al., 2005). The different animal models of *Syngap1^−/+^* are extensively discussed in a recent review (Ogden et al., 2015).

Due to a rapid increase in the availability of the types of genetically modified mice (Branchi et al., 2003), it is critical to meticulously characterize their biochemical, pathological and behavioral features and compare them with human phenotypes (Bailey et al., 2006; Crawley, 2008). Generally, laboratories involved in testing the phenotypes of genetically modified mice subject them to a battery of behavioral features to assess cognitive, motor, and sensory functions. In addition, to consider a genetically modified mouse as a disease model, the transgenic animal must fulfil at least two levels of validity to judge its psychopharmacology (van der Staay et al., 2009). An animal model should score high on the following validities: *face validity*, i.e., resemblances of behavioral phenotypes of mouse model to that of human disorder; *construct validity*, i.e., closely reconstructs and mimics the underlying cause of the disease or disorder; and *predictive validity*, i.e., drug treatments alleviate symptoms in mouse and human. A mouse model should fulfil at least face and construct validity. Indeed, various mouse models of *Syngap1^−/+^* mice satisfied face validity (Komiyama et al., 2002; Kim et al., 2003; Guo et al., 2009; Muhia et al., 2010). These various *Syngap1^−/+^* mouse models recapitulated many of the phenotypes observed in humans. For example, Komiyama et al. (2002) were the first to observe learning and memory deficits in Syngap1^−/+^ mice. Using a different model described by Kim et al. (2003), *Syngap1^−/+^* mice displayed altered social/conspecific interaction, abnormal spatial working memory, decreased anxiety-related response, hyperactivity, impaired cued conditioning behavior, increased startle reflex, increased horizontal stereotypic behavior and reduced prepulse inhibition, as well as learning deficits (Guo et al., 2009). Later, using another genetic model of *Syngap1^−/+^* mice (Muhia et al., 2010), Muhia et al. observed cognitive dysfunctions similar to the Guo et al. (2009) study. As mentioned previously, epilepsy is a prominent clinical feature observed in *SYNGAP1* patients. Accordingly, *Syngap1^−/+^* mice are prone to audiogenic seizures and have reduced seizure threshold and altered electroencephalogram (EEG; Clement et al., 2012; Ozkan et al., 2014). Therefore, based on these studies, it is clear that mouse models of *Syngap1^−/+^* (Ogden et al., 2015) mutation phenocopy the deficits observed in *SYNGAP1* patients, thereby allowing a better understanding of *Syngap1^−/+^* mutation in neuronal function and its consequence in ID.

## Role of *Syngap1* in Neurological Pathway

**Figure 3:**
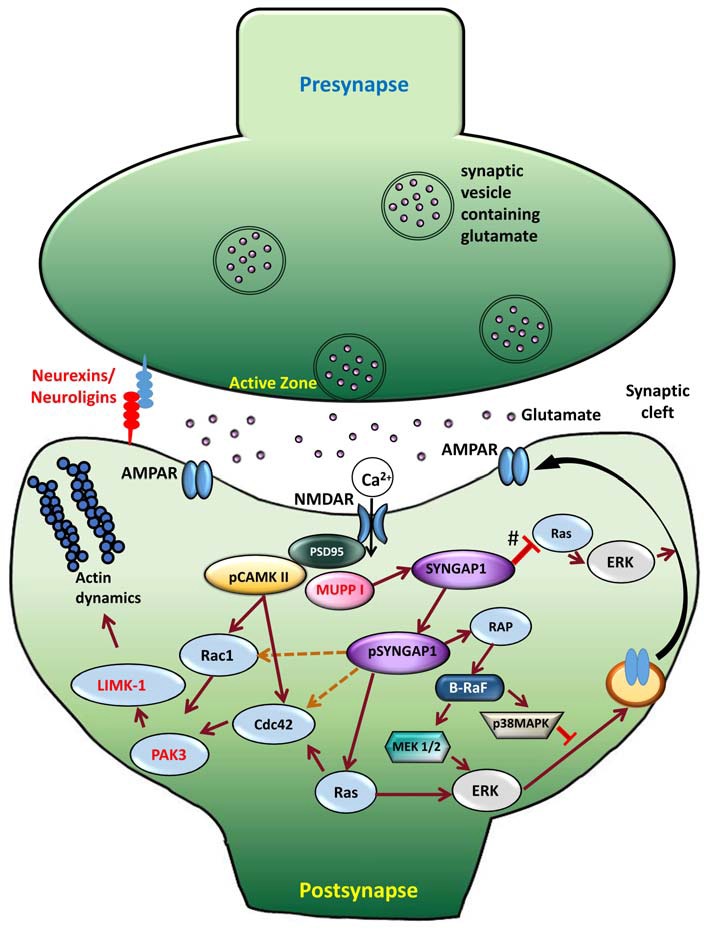
**Signaling mechanism upon phosphorylation of SYNGAP1.** Schematic model of the cellular events that link CaMKII activity to phosphorylation of SYNGAP1 and its regulation of downstream molecules. Glutamate receptors, such as NMDAR and AMPAR, are clustered at the postsynaptic active zone with a dense matrix called PSD. Upon NMDAR activation, Ca^2+^ enters the postsynaptic cytosol, triggering phosphorylation of CaMKII, which in turn phosphorylates SYNGAP1 (pSYNGAP1). pSYNGAP1 regulates Ras-GTPases controlling actin dynamics and AMPARs insertion into the postsynaptic membrane. In *Syngap1* Heterozygous mutation, the inhibition of Ras activation by SYNGAP1 (shown as #) is lost, which increases Ras activity, thereby increasing AMPAR exocytosis to the postsynaptic membrane. Phosphorylation of SYNGAP1 by cyclin-dependent kinase 5 (CDK5) activates Rap1 that increases endocytosis of AMPAR. It is not clear how pSYNGAP1 regulates other SYNGAP1-associated proteins such as Cdc42, Rac1 (dotted orange lines), which are yet to be studied.

For nearly two decades, neuroscientists have studied SYNGAP1-related signaling pathways. Synapses are extremely ordered structures that facilitate the transmission of information from presynaptic terminal to the postsynaptic membrane and, subsequently, activate signal transduction cascades that lead to suitable cellular events. In the postsynaptic membrane, two major ionotropic glutamate receptor subtypes are present—NMDARs and AMPARs. NMDARs are glutamate-sensitive ion channels that open up when glutamate and its co-agonist are bound to them. However, the actual permeation of ions through NMDAR channels occurs after the removal of Mg^2+^ block achieved by depolarization of the postsynaptic membrane. This depolarization is induced by glutamate binding to AMPARs. Subsequently to activation of NMDARs in the postsynaptic membrane, Ca^2+^ enters the dendritic spine, triggering activation of kinase cascades and thereby mediating various synaptic functions (Fan et al., 2014). NMDARs are an integral component of the PSD and bind to several PSD-enriched scaffold and signaling molecules, resulting in creation of a vast protein complex (Niethammer et al., 1996; Kennedy, 1997; Xu, 2011). This NMDAR-PSD protein complex is believed to play a vital role in the precise tuning of synapses in response to changing input stimuli pattern (Grant and O’dell, 2001; Yashiro and Philpot, 2008). SYNGAP1, one of the most abundant proteins in the PSD, is associated with NMDAR protein complex (Figure 3), which was first shown by Chen et al. (1998) and followed by Kim et al. (1998). Establishing SYNGAP1’s role in the NMDAR-mediated protein complex and signaling cascade is important to further our understanding of the etiology of SYNGAP1-mediated ID and ASD (Figure 3). SYNGAP1 has been shown to co-immunoprecipitate with PSD-95 protein complex from deoxycholate-solubilized mouse brain membrane preparations (Chen et al., 1998; Kim et al., 1998).

The GAP domain of SYNGAP1 is homologous to that of p120GAP and neurofibromin, two canonical Ras-GAPs that do not regulate Rap (Chen et al., 1998; Kim et al., 1998). However, SYNGAP1 has been shown to regulate Rap-GTPase more potently than Ras-GTPase (Pena et al., 2008). A recent study by Walkup et al. (2015) has shown that recombinant SYNGAP1 lacking 102 residues at the N-terminus is phosphorylated by cyclin-dependent kinase 5 (CDK5), as well as by CaMKII. Interestingly, phosphorylation of SYNGAP1 by CDK5 and CaMKII increases overall SYNGAP1 activity, but also alters the ratio of its GAP activity towards Ras- and Rap-GTPases. Phosphorylation of SYNGAP1 by CaMKII increases its Ras-GAP activity by 25% and its Rap-GAP activity by 76%. CDK5 increases recombinant SYNGAP1 activity on Ras-GAP by 98% and its Rap-GAP activity by 20%. Furthermore, upon NMDAR stimulation, Ca^2+^ entering the synapse dissociates CaMKII from SYNGAP1 and phosphorylates SYNGAP1 (pSYNGAP1). This leads to activation of Ras that activates proteins downstream, consequently to AMPAR insertion in the postsynaptic membrane. Therefore, the phosphorylation of SYNGAP1 is believed to be important in regulating transient changes in the number of surface AMPA receptors or gradually adjusting their steady-state level.

By biochemical analysis of proteins containing the GAP, Krapivinsky et al. (2004) have identified the C2 domain as essential for the Rap-GAP activity of SYNGAP1, which is in line with the recent observation (Walkup et al., 2015). The homology of SYNGAP1 with other Ras-GAP domains across species and *in vivo* associations of SYNGAP1 with the NMDA receptor complex indicate that SYNGAP1 plays a role in Ras-mediated signaling in excitatory synapses, particularly in response to Ca^2+^ (Kim et al., 1998; Carlisle et al., 2008). Apart from regulating Ras-mediated signaling, SYNGAP1 has been shown to mediate the activity of other major signaling proteins, such as Rac, p-Cofilin, p21-activated kinase (PAK) and LIMK (Carlisle et al., 2008) and these proteins were shown to be elevated at basal conditions in *Syngap1^−/+^* mice.

## Role of *Syngap1* in Regulating Dendritic Spine Morphology and Plasticity

The neuronal signaling cascades underlying synaptic plasticity and dendritic spine structure have been intensely studied and a multitude of signaling molecules have been identified (Kennedy et al., 2005; Patterson and Yasuda, 2011). Initial stages of dendritic spine formation and neuronal connections depend on cytoskeleton protein, F-actin, which is regulated by Ras- and Rac-GTPases. Therefore, it is possible that the dendritic spine structure and function would be altered in *Syngap1^−/+^* mutations, which may explain the behavioral deficits observed in *Syngap1^−/+^* patients.

Ras- and Rac-mediated signaling cascade, including ERK and MAPK, has also been shown to play a major role in normal synaptic transmission and in long-term potentiation (LTP; Carlisle and Kennedy, 2005; Kennedy et al., 2005) by modulating insertion of AMPARs into the postsynaptic membrane (Figure 3). On the contrary, opposite effects were observed in *Syngap1* knockout in neuronal cultures using small interfering RNA. Studies from neuronal culture have demonstrated that overexpression of *Syngap1* resulted in a remarkable down regulation of AMPAR-mediated currents (Rumbaugh et al., 2006). In contrast, AMPAR-mediated currents were increased in *Syngap1^−/+^* in neuronal cultures treated with small interfering RNA.

Under basal conditions, when fEPSPs, which are predominantly mediated by AMPARs, were measured from adult mouse CA1 hippocampal pyramidal region, *Syngap1^−/+^* mice did not show any abnormal excitatory synaptic responses (Komiyama et al., 2002). Furthermore, the presynaptic fibers were required to evoke an equivalent postsynaptic response in slices from wild type (WT), and *Syngap1^−/+^* mouse responses were not altered suggesting that the activity of postsynaptic AMPARs were unchanged. However, these experiments were performed in adults and the genes encoding the proteins that regulate synaptic function reach their peak level of expression during early stages of development (State and Levitt, 2011; State and Sestan, 2012). Indeed, *Syngap1* Het mutations can affect synaptic transmission during early developmental period. Thus, Vazquez et al. (2004) have reported that the *Syngap1^−/+^* mice form dendritic spines and synapses prematurely, and that spines ultimately become larger in *Syngap1^−/+^*. In addition, Clement et al. (2012, 2013) have shown elevated input-output relationship in extracellular field recording and AMPAR-mediated mEPSCs, which reached WT adult level 2 weeks after birth (PND14), confirming the earlier findings (Vazquez et al., 2004). These studies suggest that SYNGAP1 controls the trajectory of synapse maturation during a particular period of development by controlling protein synthesis and homeostatic synaptic plasticity during development (Wang et al., 2013).

In hippocampal pyramidal neurons, spine structure is tightly correlated with synaptic function (Noguchi et al., 2011). *Syngap1^−/+^* mutation disrupts proper development of dendritic spine structures. *Syngap1^−/+^* mice have more mature, mushroom-shaped spines during early stages of development (PND14) suggesting precarious prematuration of dendritic excitatory spine structures (Clement et al., 2012, 2013). Further, accelerated spine formation and premature spine pruning have been observed in developing neocortical tissue of *Syngap1^−/+^* mice (Aceti et al., 2015). These abnormalities observed during development in *Syngap1^−/+^* mutation persisted into adulthood (Vazquez et al., 2004; Carlisle et al., 2008; Clement et al., 2012), consistent with the spine dysfunction theory of cognitive disorders.

Altered maturation of dendritic spine morphology and function can lead to various learning and memory deficits (Peca et al., 2011; Goncalves et al., 2013). Indeed, patients with *SYNGAP1* Het mutations were observed to have learning and memory deficits (Hamdan et al., 2009, 2011a). To understand the impact of early maturation of dendritic spine morphology and function on learning and memory in *Syngap1^−/+^* mice, synaptic plasticity studies were carried out by various labs. There are two major forms of synaptic plasticity, LTP and LTD (long term depression), which have been considered to represent the cellular correlates of learning and memory and are both dependent on local protein synthesis (Volianskis et al., 2015). Deficits in LTP have been observed in many animal models of ID and ASD (Volk et al., 2007, 2015; Pavlowsky et al., 2012). Alterations in signaling proteins function can lead to anomalous synaptic plasticity and dendritic spine structure and can correlate with cognitive impairments in patients with ID and ASD (McKinney, 2005; Penzes et al., 2011; van Bokhoven, 2011; Kroon et al., 2013). Interestingly, adult *Syngap1^−/+^* mice do show reduced LTP in the CA1 hippocampal region, which is likely due to reduced activation of Ras and ERK during LTP (Komiyama et al., 2002; Kim et al., 2003; Ozkan et al., 2014), suggesting that reduced level of SYNGAP1 derepresses the resting levels of activated Ras and ERK. Additionally, SYNGAP1 has been shown to rapidly disperse from spines during and after LTP because of the phosphorylation of SYNGAP at Ser1108/1138 by CaMKII. Subsequently, SYNGAP1 activates Ras, which triggers long-term changes in spine size, suggesting the inhibition of stable LTP by SYNGAP1 (Araki et al., 2015).

## Critical Period of Plasticity

**Figure 4:**
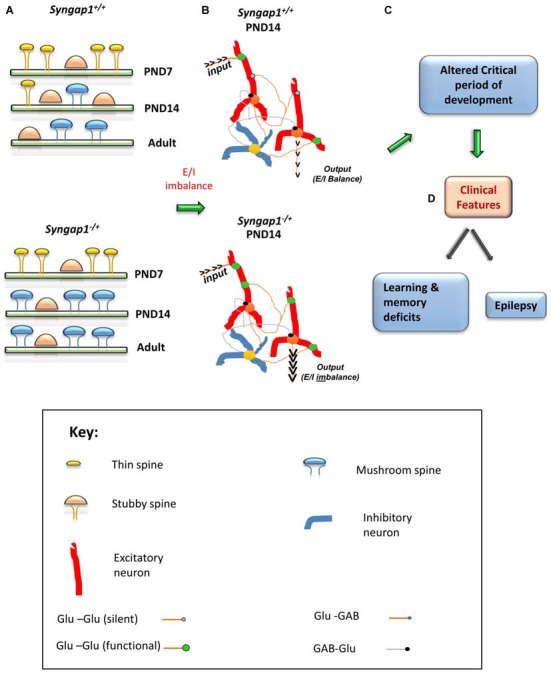
**Schematic model of the impact of *Syngap1* haploinsufficiency on neuronal circuit function.** This figure illustrates the impact of *Syngap1* heterozygous mutation on dendritic spine morphology, neuronal connection organization, and behavioral phenotypes. Heterozygous mutations in *Syngap1* lead to prematuration of dendritic spine morphology during early stages of development **(A)**. This causes abnormal formation and elimination of spines that leads to altered spine density and excitatory neuronal connections during development in the cortex (Aceti et al., 2015). Further, the abnormal cortical excitatory neuronal connections lead to E/I imbalance during early stages of development, which persists, into adult stages in *Syngap1* Hets **(B)**. Consequently, these abnormalities bring about altered duration of critical period of development **(C)**, which leads to cognitive and social dysfunction **(D)**. PND, Post-natal Day. The gene products implicated in intellectual disability (ID) and/or autism spectrum disorder (ASD) are marked in Red color text. Some features are modified with permission based on Clement et al. (2012).

Steady increase of synaptic AMPARs and subsequent functional unsilencing of glutamatergic inputs are characteristics of early postnatal development (Kerchner and Nicoll, 2008). Premature acquisition of functional AMPARs during development is suggestive of an acceleration of neurodevelopmental pattern during a critical period of development. A critical period is a regulated time window during which the sensory experience and intrinsic neuronal activity provide information that are essential for normal development and refinement of neuronal circuits (Meredith et al., 2012). Any alteration to dendritic spine structure and function during this critical period can have a lasting effect on cognitive functions, the development of which requires the formation and refinement of synaptic networks of neurons in the brain. Precariously high AMPAR/NMDAR ratios observed in *Syngap1^−/+^*mice could lead to altered duration of plasticity-related critical periods. In the thalamocortical pathway, generation of LTP becomes difficult towards the end of the first postnatal week (Crair and Malenka, 1995). However, high frequency stimulation failed to elicit LTP in PND4 and PND7 in *Syngap1^−/+^* mice, while LTP was generated in PND4 WT animals. Given that SYNGAP1 has been shown to suppress AMPAR insertion in the postsynaptic membrane, the main explanation for LTP failure at synapses would be a precocious unsilencing of the developing thalamocortical pathway. Indeed, *Syngap1^−/+^* mice have altered unsilencing of post-synapses during early stages of development in the thalamocortical pathway (Clement et al., 2013) and altered formation and elimination of dendritic spines (Aceti et al., 2015). These studies further confirm the hypothesis that prematuration of dendritic spine structure due to accumulation of AMPAR at synapses shortens the duration of the critical window of plasticity leading to altered behavioral function (Figure 4).

In addition to LTP, another form of synaptic plasticity, LTD is important for proper formation and refinement of neuronal connections (Feldman and Knudsen, 1998; Hensch, 2005). Thus, it would be predicted that thalamocortical synapses exhibits LTP as well as LTD. Similar to LTP, the amount of LTD induced in thalamocortical connections exhibited a developmental reduction with little or no depression remaining by P10–12 (Feldman et al., 1998). This signals the end of critical period of plasticity in thalamocortical synapses. In addition, this study suggests that NMDAR dependent LTD modulates the efficacy of synapses previously unsilenced by LTP (Daw et al., 2007), thereby, allowing the synapses to modulate the connections based on the input specific activity. However, it is not clear LTD can induce resilencing of functional synapses during critical period of development. Given the importance of LTD in critical period of plasticity, it is not clear whether LTD is altered during critical period of development in *Syngap1^−/+^*mice.

## Proposed Model of *Syngap1^−/+^* Mutation in Neuronal Development and Maturation

One of the common features observed in most forms of ID is the inability to develop and maintain normal dendritic spine architecture and proper function at synapses, which lead to abnormal neuronal connections. Based on the above discussion, it is evident that *Syngap1^−/+^* mutations lead to abnormal dendritic spine maturation during development. All mutations in *Syngap1* are predicted to truncate the protein, thus decreasing the ability of SYNGAP1 to bind to the molecules downstream in the signaling pathway. Phosphorylation of SYNGAP1 is regulated by CaMKII, which reduces SYNGAP1’s control of Ras-GTPase, leading to Ras activation by increasing the GTP-bound form of Ras. Further, phosphorylation of SYNGAP1 by CaMKII increases the ratio of Rap1-GAP to Ras-GAP (Walkup et al., 2015). This would shift the steady-state balance of AMPAR trafficking at the synapse towards exocytosis by decreasing the level of active Rap1 compared to active Ras, which would result in an increased surface AMPAR. In contrast, phosphorylation of SYNGAP1 by CDK5 decreases its ratio of Rap1-GAP to Ras-GAP activity, which would allow more AMPAR to be endocytosed from the postsynaptic surface. Therefore, SYNGAP1 negatively regulates Ras activation and insertion of AMPA receptors in the postsynaptic membrane. Phosphorylation of SYNGAP1 creates transient changes in the number of AMPARs and gradually adjusts the steady-state level of AMPARs in the postsynaptic membrane (Figure 3). However, due to *Syngap1^−/+^* mutation, truncated SYGNAP1 fails to inhibit Ras activity, thereby facilitating conversion of inactive, GDP-bound Ras to an active, GTP-bound form and increasing the level of Ras activation. Ras is one of the important components of the signaling pathway underlying NMDA receptor mediated activation of ERK (Iida et al., 2001). Thus, increase in Ras activity elevates the level of phosphorylated ERK, which further facilitates the insertion of AMPARs to the postsynaptic membrane (Derkach et al., 2007). In this aspect, SYNGAP1 is a key molecule that facilitates a cross talk between CaMKII and Ras/MAPK signaling pathways that leads to AMPAR trafficking, thereby controlling the excitatory synaptic strength, particularly in developing neurons. However, SYNGAP1’s control of excitatory synaptic strength during development is lacking in ID patients or in the *Syngap1^−/+^* mice model.

Furthermore, increased level of Ras activation leads to activation of LIMKII, CDC42 and p-cofilin, which regulate actin cytoskeleton (Figure 3). Actin is the major cytoskeletal element in dendritic spines, where it serves both as framework for the spine structure and as a scaffold for postsynaptic proteins (Dillon and Goda, 2005). Cofilin is best known as a regulator of actin whose assembly and disassembly depends upon the concentration of cofilin. In *Syngap1^−/+^* mice, increased levels of p-Cofilin shift the equilibrium towards the more stable actin form, F-actin. This makes the dendritic spine more stable at an early stage of development. Thus, more stable form of actin combined with an increase in insertion of AMPAR into the postsynaptic membrane during development (PND14–16 in hippocampus in Hets) causes the dendritic spines to mature into mushroom shaped spines, which occurs earlier in *Syngap1^−/+^* than in WT animals (Figure 4).

This leads to elevated excitatory synaptic transmission causing Excitatory/Inhibitory (E/I) imbalance, particularly during the critical period of development. Due to the altered E/I balance, humans, as well as *Syngap1^−/+^* mice, are prone to epileptic seizures. The altered E/I balance observed in *Syngap1^−/+^* mice is representative of an altered form of synaptic homeostasis that degrades the ability of mature neurons to optimally balance excitation relative to inhibition. Indeed, truncation of SYNGAP1 occludes the ability of neurons to scale up synaptic strength in response to activity, suggesting that SYNGAP1 associated signaling is necessary for maintaining homeostatic synaptic plasticity (Wang et al., 2013). In fact, altered dendritic spine morphology and function during the critical period of development causes a coordinated acceleration of dendritic elongation, spine formation, and elimination (pruning) in cortical neurons, which may result in altered neuronal connectivity and abnormal closure of critical period plasticity (Figure 4). Interestingly, E/I imbalance in *Syngap1^−/+^* mutation leads to altered pruning of spines, which in turn causes abnormal connections (non-target) in neurons and negatively affects the organization of neuronal circuits (Aceti et al., 2015). The abnormal pruning of spines and connections between neurons could be a consequence of an altered duration of critical period observed in *Syngap1^−/+^* mice. This would prevent the neuronal connections to be actively refined by the surrounding environment in which the individual exists.

There appear to be independent critical periods of development for different modalities, ranging from basic visual processing to language and social skills, which are observed to be affected in patients with *SYNGAP1* heterozygous mutation. *Syngap1^−/+^* mice displayed early closure of critical period of plasticity during development (Clement et al., 2013). The precise development of the timing of critical periods during cortical development is essential for the proper organization of synaptic connections and neuronal circuit formation. Thus, premature closure of plasticity window during development could contribute to altered refinement of cortical circuits that persist throughout the life of an animal and thus contributing to cognitive deficits in *Syngap1^−/+^* mice. Thus, transient neurodevelopmental events induced by *Syngap1* mutations could cause life-long disruptions to cognition and behavior that are difficult to treat in adulthood.

## Role of astrocytes in ID and ASD

While neurons are considered as major players in brain function such as perception, social behavior and memory, astrocytes have been relegated to a far lesser supporting role. However, in recent years, emerging evidences suggest that signaling between astrocytes and neurons at the tripartite synapse plays an important role during the critical period of development (Stevens, 2008; Clarke and Barres, 2013). Although astrocytes were considered to play a passive onlooker in the synapse, but studies show that, they are necessary for neuronal maturation, function, and development of neurons. During early stages of development, astrocytes and neurons are formed from neuronal precursor cells (Freeman, 2010). Three-dimensonal reconstructions of dye-filled astrocytes reveal that astrocytes extend thousands of intricate processes that are organized into large, non-overlapping anatomical domains. It has been estimated that a single astrocyte can associate with multiple neurons and over 100,000 synapses (Bushong et al., 2002; Halassa et al., 2007a,b). While astrocytes are incapable of generating action potentials, they do secrete a wide array of gliotransmitters and express many of the same channels, receptors and cell surface molecules similar to neurons (Haydon, 2000; Fields and Stevens-Graham, 2002; Fiacco and Mccarthy, 2006).

Neurons rely on astrocytes to instruct the formation and elimination of their synapses lead to the possibility that astrocytes work in parallel with and interacts with, the neuronal processes that control circuit formation. One of the first evidences that astrocytes contribute majorly in critical period of development came from a study by Muller and Best (1989) that injection of immature astrocytes into the adult visual cortex reopened the window of ocular dominance plasticity. Further, a study from purified rodent ganglion cells (RGCs) suggested that RGCs formed very few syanpses in the absesnce of astrocytes. However, when cultured in the presence of astrocytes, or in a medium that had been conditioned with any other soluble signals released by astrocytes, RGCs can form ten-fold more excitatory synapses and synaptic functionality was increased (Pfrieger and Barres, 1997; Ullian et al., 2001). Not only astrocytes regulate the development, maturation, and function of excitatory neurons, they are a requisite for the development of inhibitory synapses. Liu et al. (1996) showed that local contact between neurons and astrocytes significantly increased the amplitude and density of GABA_A_ receptors in developing hippocampal neurons. In addition, astrocytes were shown to regulate chloride ion gradient in cultured spinal cord neurons and convert GABAergic neurons from excitatory to inhibitory (Li et al., 1998). These studies suggest that immature astrocytes are necessary for critical period of development and it is linked to maturation of astrocytes.

It is evident from the above mentioned studies that astrocytes play a major role in normal neuronal development and function, it would not be surprising that astrocytes contribute in some capacity to almost all pathological conditions of the nervous system (Lin and Koleske, 2010; Parpura et al., 2012). Consequently, astrocyte-dysregulated function has been linked with the progressive pathology of ischemic stroke, epilepsy and to a number of neurodegenerative disorders including amyotrophic lateral sclerosis, Huntington’s disease, Parkinson’s disease, Rett syndrome, FXS, and autism (Yamamuro et al., 2015). FXS is one of the most common form of ID and affects 1 in 4000 males and 1 in 6000 females. Fragile X mental retardation protein (FMRP) is reported to be expressed in Oligodendritic precursor cells but not mature oligodendrocytes (Wang et al., 2004). However, a study by Pacey and Doering (2007) reported expression of FMRP in astrocytes. Further, they showed that WT neurons grown on *Fmr1* KO astrocytes exhibited significantly altered dendritic arbor morphologies, whereas *Fmr1* KO neurons cultured with WT astrocytes, the alterations in dendritic morphologies and synaptic protein expression were prevented (Jacobs and Doering, 2010; Jacobs et al., 2010). These experiments were the first to suggest that astrocytes contribute to the normal development of dendritic spine morphology and function. Therefore, it is important to study the role of astrocytes in *Syngap1^−/+^* mutations. However, there are no studies to date to suggest expression of *Syngap1* in astrocytes or its role in ID due to *Syngap1^−/+^*.

## Conclusion

Basic research in ID and ASD using model organisms has been critical in advancing our understanding of many NDDs. Important insights into the neurophysiology of *Syngap1^−/+^* mutations, especially the regulation of dendritic spine formation and function, has been gained from the study of *Syngap1^−/+^* mouse models. Although it is clear from these studies that SYNGAP1 is a negative regulator of AMPAR insertion in the postsynaptic membrane that regulates dendritic spine structure and function, certain questions still remain unanswered, such as which downstream proteins are regulated by affected by *Syngap1^−/+^* mutations. The other major question is to find the precise window during development to address the symptoms observed in ID. In fact, repairing pathogenic *Syngap1^−/+^* mutation after the end of critical period of development failed to rescue neurophysiological and cognitive functions. Therefore, it is important to find the right period of development in order to rescue the cognitive deficits observed in *Syngap1^−/+^* mutation. One of the means of finding therapeutic targets is to find a protein which has been implicated in another ID and ASD that produces similar or opposite cellular and behavioral phenotypes as that of *Syngap1^−/+^* mutants. The opposing effects of these mutations may balance one another at synaptic and behavioral function (Auerbach et al., 2011). Understanding the effect of complementary pathways to rescue a gene of interest, for example *Syngap1^−/+^* mutation, would allow better therapeutic designs to alleviate ID symptoms (earlier the better). It is important to understand where an ID and ASD patient lies on the spectrum of synaptic and behavioral dysfunction to choose an appropriate therapy. Thus, continued study of various disorders that exhibit ID and ASD phenotypes may lead to better therapeutic targets.

## Author Contributions

All authors listed, have made substantial, direct and intellectual contribution to the work, and approved it for publication.

## Conflict of Interest Statement

The authors declare that the research was conducted in the absence of any commercial or financial relationships that could be construed as a potential conflict of interest.

## Footnotes

^1^http://www.ncbi.nlm.nih.gov/gene/8831

^2^http://www.ncbi.nlm.nih.gov/gene/240057
